# Brain-Derived Neurotrophic Factor Inhibits the Function of Cation-Chloride Cotransporter in a Mouse Model of Viral Infection-Induced Epilepsy

**DOI:** 10.3389/fcell.2022.961292

**Published:** 2022-07-08

**Authors:** Dipan C. Patel, Emily G. Thompson, Harald Sontheimer

**Affiliations:** ^1^ Glial Biology in Health, Disease, and Cancer Center, Fralin Biomedical Research Institute at Virginia Tech-Carilion, Roanoke, VA, United States; ^2^ Department of Neurobiology, University of Alabama at Birmingham, Birmingham, AL, United States; ^3^ School of Neuroscience, Virginia Tech, Blacksburg, VA, United States

**Keywords:** brain-derived neurotrophic factor (BDNF), potassium chloride co-transporter-2 (KCC2), Theiler’s murine encephalomyelitis virus (TMEV), infection, inflammation

## Abstract

Well over 100 different viruses can infect the brain and cause brain inflammation. In the developing world, brain inflammation is a leading cause for epilepsy and often refractory to established anti-seizure drugs. Epilepsy generally results from an imbalance in excitatory glutamatergic and inhibitory GABAergic neurotransmission. GABAergic inhibition is determined by the intracellular Cl^−^ concentration which is established through the opposing action of two cation chloride cotransporters namely NKCC1 and KCC2. Brain-derived neurotrophic factor (BDNF) signaling is known to regulate expression of KCC2. Hence we hypothesized that viral induced epilepsy may result from aberrant BDNF signaling. We tested this hypothesis using a mouse model of Theiler’s murine encephalomyelitis virus (TMEV) infection-induced epilepsy. We found that BDNF levels in the hippocampus from TMEV-infected mice with seizures was increased at the onset of acute seizures and continued to increase during the peak of acute seizure as well as in latent and chronic phases of epilepsy. During the acute phase of epilepsy, we found significant reduction in the expression of KCC2 in hippocampus, whereas the level of NKCC1 was unaltered. Importantly, inhibiting BDNF using scavenging bodies of BDNF in live brain slices from TMEV-infected mice with seizures normalized the level of KCC2 in hippocampus. Our results suggest that BDNF can directly decrease the relative expression of NKCC1 and KCC2 such as to favor accumulation of chloride intracellularly which in turn causes hyperexcitability by reversing GABA-mediated inhibition. Although our attempt to inhibit the BDNF signaling mediated through tyrosine kinase B–phospholipase Cγ1 (TrkB-PLCγ1) using a small peptide did not change the course of seizure development following TMEV infection, alternative strategies for controlling the BDNF signaling could be useful in preventing seizure generation and development of epilepsy in this model.

## Introduction

Epilepsy is a devastating neurological disorder affecting around 50 million people worldwide showing a higher prevalence in low/middle-income countries (LMIC) (Beghi, 2020). In these countries in particular, viral infection of the central nervous system (CNS) is a common cause of acquired temporal lobe epilepsy (TLE) for which there is currently no effective antiepileptic treatment for patients at risk (Patel and Wilcox, 2017; Löscher and Howe, 2022). Over two dozen antiseizure drugs are clinically approved that target primarily excitatory and inhibitory neurotransmission. However, almost one-third of the epilepsy patients remain pharmacoresistant. Several molecular mechanisms of epileptogenesis involving alternative molecular pathways regulating the excitation-inhibition balance have been proposed. One of these mechanisms involves brain-derived neurotrophic factor (BDNF) signaling through its major cognate receptor tyrosine kinase B (TrkB).

BDNF is a growth factor belonging to the neurotrophin family and mediates diverse functions through TrkB. Given its robust effects on neuronal development and differentiation, and synaptic plasticity, perturbations in the BDNF signaling has been reported in many neurological conditions including epilepsy (Park and Poo, 2013; Sasi et al., 2017). Earlier studies found that electric kindling-induced seizures caused an increase in gene expression of BDNF in cortical and hippocampal neurons (Ernfors et al., 1991). Similar increase in the induction of BDNF mRNA was also correlated with seizures induced by a systemic administration of kainic acid in rats (Dugich-Djordjevic et al., 1992). Increasing the BDNF signaling either by direct infusion of BDNF in hippocampus or by genetically overexpressing BDNF or TrkB in mice increased seizure severity or susceptibility, or induced spontaneous limbic seizures (Croll et al., 1999; Scharfman et al., 2002; Lahteinen et al., 2003; Xu et al., 2004). In contrast, kindling-induced epileptogenesis was markedly suppressed in mice with a heterozygous deletion of BDNF gene (Kokaia et al., 1995) and in mice infused intraventricularly with the recombinant TrkB receptor bodies that neutralize BDNF (Binder et al., 1999). Collectively these studies suggested that increased activation of TrkB through BNDF underlies network hyperexcitability and epileptogenesis. Further studies supported this conclusion by demonstrating reduction in seizures and epileptogenesis in mice using genetic and pharmacological tools designed to inhibit the BDNF-TrkB signaling (He et al., 2004; Liu et al., 2013; Gu et al., 2015).

BDNF-mediated hyperexcitability is partly caused by its inhibitory effects on fast GABAergic inhibition, which in turn, is maintained by low intracellular chloride concentration ([Cl^−^]_i_) (Doyon et al., 2016; Porcher et al., 2018). Two cation-chloride cotransporters, the K^+^-Cl^−^ cotransporter 2 (KCC2) and the Na^+^-K^+^-Cl^−^ cotransporter (NKCC1), are responsible for shuttling Cl^−^ in and out of the cell, respectively (Doyon et al., 2016). An increase in the ratio of NKCC1/KCC2 can increase [Cl^−^]_i_ and cause depolarizing excitatory shift in GABAergic activity. Furthermore, loss-of-function mutations in KCC2 are causally linked with human infantile epilepsy and KCC2 impairment have been found in patients with idiopathic and acquired epilepsy (Moore et al., 2017). Importantly, BNDF has been shown to impair fast GABAergic inhibition by reducing the cell surface expression of KCC2 and raising the intracellular Cl^−^ level (Rivera et al., 2002; Ferrini et al., 2013).

The present study was performed to question whether BDNF-TrkB signaling contributes to hyperexcitability by altering the activity of cation-chloride cotransporters in a model of Theiler’s murine encephalomyelitis virus (TMEV) infection-associated acquired limbic epilepsy. We found a significant increase in the protein levels of BDNF in hippocampus after TMEV infection that was coincident with seizure activity. Further, a decrease in the level of hippocampal KCC2 was correlated with peak acute seizures. Importantly, neutralizing endogenously released BDNF largely restored the level of KCC2 in hippocampal slices from the mice with acute seizures. However, treatment with pY816, an inhibitor of the BDNF-TrkB-PLCγ1 signaling cascade, had no effect on acute as well as chronic seizures. In conclusion, these results suggest that increased release of BDNF may contribute to TMEV infection-induced seizures by impairing KCC2-mediated maintenance of fast GABAergic inhibition.

## Materials and Methods

### Animals

C57BL/6J (strain # 000664) and BDNF^+/−^ mice (strain # 002266) were purchased from the Jackson laboratories, United States. Both male and female mice aged 7–9 weeks were included in all the experiments conducted. BDNF^+/−^ mice are on a C57BL/6J genetic background, and they were genotyped to confirm the deletion of the gene. Upon arrival, mice were allowed to acclimatize for at least 3 days at our facility before conducting the experiments. Mice were housed in groups with maximum five mice per cage in an environmentally controlled vivarium room providing 12 h of light and dark cycles starting at 6:00 a.m. Food and water were freely accessible and provided ad libitum. Total 104 wild-type (WT) C57BL/6J and 10 BDNF^+/−^ mice were used in the present study. A variation of block randomization method was used to assign a block of two mice randomly to either treatment or control group. All the procedures performed were in accordance with the guidelines provided and approved by the Institutional Animal Care and Use Committee of Virginia Tech.

### Procedure of TMEV Infection and Handling-Induced Seizure Monitoring

A Daniel’s strain of Theiler’s murine encephalomyelitis virus (TMEV) was used to induce seizures in mice. TMEV was kindly provided by the laboratories of Drs. Karen S. Wilcox and Robert S. Fujinami from the University of Utah. The titer of the stock used was 2 × 10^7^ plaque forming units (PFU) per ml.

Mice were briefly anesthetized with 3% isoflurane and injected with 20 µl of either phosphate-buffered saline (PBS) or TMEV solution (2 × 10^5^ PFU (biochemical studies) or 3.5 × 10^5^ PFU (electrocorticography)) intracortically (i.c.) in the right hemisphere by inserting a 28-gauge needle perpendicular to the skull surface. The head surface was disinfected with 70% isopropyl alcohol swab and the injection site was approximated slightly medial to the equidistant point on the imaginary line connecting the right eye and the right ear. A sterilized syringe containing a plastic sleeve on the needle to expose only 2.5 mm of needle from the tip was used to restrict the injection in a cortical region about 2–3 mm lateral and 1–2 mm posterior to bregma without damaging the hippocampus. The needle was kept in place undisturbed for about 1 min after injection and retracted slowly to minimize leakage. The injury site was disinfected post-injection. Mice resumed their normal behavior within 5–10 min of the procedure.

TMEV-infected mice experience handling-induced acute behavioral seizures typically between 2–8 days post-infection (dpi) (Patel et al., 2017). Mice were briefly agitated by shaking their cage and observed for behavioral seizures for about 5 min in two observation sessions daily separately by at least 2 h. Most of the acute seizures occurred within a minute of handling the mice. Seizure severity was graded using a modified Racine scale as follows: stage 1, mouth and facial movements; stage 2, head nodding; stage 3, forelimb clonus; stage 4, forelimb clonus, rearing; stage 5, forelimb clonus, rearing, and falling; and stage 6, intense running, jumping, repeated falling, and severe clonus (Patel et al., 2019b).

### Tissue Collection for Protein Analysis

Since most of the TMEV-infected mice developed handling-induced behavioral seizures, all the biochemical studies were conducted in TMEV-infected mice that showed acute seizures. TMEV- and sham-treated mice were sacrificed at 1, 3, 5, 14 and 60 dpi depending on the experiment. The ipsilateral and contralateral hippocampi were rapidly dissected out and collected separately. All the tissue samples were flash-frozen using 2-methylbutane chilled on dry ice and stored at –80°C until further processing.

### Enzyme-Linked Immunoassay

Mature BDNF was measured in the hippocampal lysate using a rapid ELISA kit (BEK-2211, Biosensis) according to the manufacturer’s instructions. Briefly, ipsilateral and contralateral hippocampi were homogenized in an acid extraction buffer (50 mM sodium acetate, 1 M sodium chloride, 0.1% Triton X-100, pH 4.0 using glacial acetic acid) in a 1:10 weight-by-volume ratio (10 µl buffer/mg of tissue) using a rotor-stator homogenizer. The homogenate was centrifuged (13,000 g, 30 min, 4°C), and the supernatant was collected. Since BDNF is bound to its receptors and chaperons within tissues, it is difficult to detect it by ELISA. However, extraction under acidic condition releases bound BDNF and precipitates its receptors, so that it can be immunodetected (Kolbeck et al., 1999). Protein concentration in supernatant was determined using bicinchoninic acid (BCA) assay (Pierce™ BCA Protein Assay Kit, Catalog #23225, ThermoFisher Scientific), and appropriate amount of supernatant was neutralized (100 mM phosphate buffer, pH 7.60) before using for ELISA. The concentration of mBDNF was determined by measuring the absorbance of final reaction product at 450 nm using a microplate reader (Epoch2, BioTek).

### Gel Electrophoresis and Western Blot

Protein levels of KCC2 and NKCC1 were quantified by Western blot analysis. The hippocampi samples were homogenized using a rotor-stator homogenizer in a lysis buffer (50 mM Tris-HCl pH 8.00, 150 mM NaCl, 1% Triton X-100, 0.5% sodium deoxycholate, 0.1% sodium dodecyl sulfate, protease inhibitors (P8340, Sigma) and phosphatase inhibitors (P0044, Sigma); 10 µl lysis buffer per mg of tissue), and the supernatant was collected after centrifugation (15,000 g, 20 min, 4°C). Total protein concentration was measured by BCA protein assay. 10–25 µg total protein sample was denatured at 50°C for 15 min and then electrophoresed using polyacrylamide gel (4–15% or 4–20% tris-glycine extended polyacrylamide gel; 567–1,085 and 567–1,095, Bio-Rad) under denaturing conditions. The proteins were transferred to a PVDF membrane, and immunodetected by measuring either chemiluminescence or fluorescence. Densitometric analysis of protein levels was performed using ImageJ (NIH) or Image Studio (LI-COR) software.

Primary antibodies used were rabbit polyclonal anti-KCC2 IgG (1–2 μg/ml; 07–432, Millipore Sigma), mouse monoclonal anti-NKCC1 IgG1 (0.5 μg/ml; T4, Developmental Studies Hybridoma Bank), and mouse monoclonal anti-α-tubulin IgG1 (0.1–0.5 μg/ml; A11126, Invitrogen). Secondary antibodies used were goat anti-mouse IgG HRP (0.3 μg/ml, 62–6520, Invitrogen), goat anti-rabbit IgG HRP (0.2 μg/ml; 65–6120, Invitrogen), IRDyer^®^ 680RD donkey anti-mouse IgG (0.05 μg/ml; 925–68072, LI-COR), and IRDyer^®^ 800CW donkey anti-rabbit IgG (0.05 μg/ml; 925–32213, LI-COR).

### BDNF-TrkB Inhibitors

Recombinant human TrkB Fc chimera protein (rhTrkB-Fc) (688-TK, R&D) (BDNF scavenging bodies) was used to inhibit BDNF-mediated signaling in acute brain slices. Recombinant human IgG1 Fc protein (rhIgG1-Fc) (110-HG, R&D) was used as a control recombinant protein for rhTrkB-Fc. To test the effect of these recombinant proteins on the protein level of KCC2, acute coronal brain slices (300 µm thick) from TMEV-infected mice with seizures and the control mice were prepared using vibratome (VT1200S, Leica) at 5 dpi in a cold N-methyl-D-glucamine (NMDG)-based slicing buffer (concentrations in mM: 130 NMDG, 1.5 KCl, 1.5 KH_2_PO_4_, 23 choline bicarbonate, 12.5 D-glucose, 0.4 L-ascorbic acid, 0.5 CaCl_2_, and 3.5 MgCl_2_; pH 7.3–7.4, osmolality 300–320 mOsm/kg). The slices were collected in artificial cerebrospinal fluid (ACSF) recovery buffer (concentrations in mM: 125 NaCl, 3 KCl, 1.25 NaH_2_PO_4_, 25 NaHCO_3_, 12.5 D-glucose, 2 CaCl_2_, and 2 MgCl_2_; pH 7.3–7.4; osmolality, 310–320 mOsm/kg) and allowed to recover from the slicing-induced surface damage in ACSF at 32°C for 30 min. The slices were then washed with ACSF to remove dead surface cells and debris and incubated in 250 ng/ml solution of rhTrkB-Fc or rhIgG1-Fc in ACSF at 32°C. All the solutions used for processing brains and brain slices were continuously oxygenated with a mixture of 95% oxygen and 5% carbon dioxide. After 4.5 h, drug solution was discarded, and the slices were washed with ACSF. The hippocampal and cortical regions were quickly dissected out and collected in vials containing lysis buffer (150 and 250 μl, respectively; recipe same as described in Western blot procedure). The samples were homogenized using a rotor-stator homogenizer and the supernatant was collected after centrifugation (15,000 g, 20 min, 4°C). Protein assay and western blot were then conducted as described above to measure the level of KCC2.

To inhibit BDNF signaling *in vivo*, a small peptide, pY816, was used. pY816 (YGRKKRRQRRR-LQNLAKASPVpYLDI) and its negative control scrambled (Scr) peptide sequence (YGRKKRRQRRR-LVApYQLKIAPNDLS) were synthesized by Tufts University peptide synthesis core facility. Mice were treated with a solution of pY816 or Scr prepared in PBS (10 mg/kg at 5 ml/kg body weight) by retroorbital intravenous (i.v.) injections once daily between 0–14 dpi starting immediately after TMEV-infection. Topical anesthetic bupivacaine was applied to the eyeballs of mice before performing retroorbital injections.

### Surgical Procedure for Video-Electrocorticography (vECoG)

**FIGURE 1:**
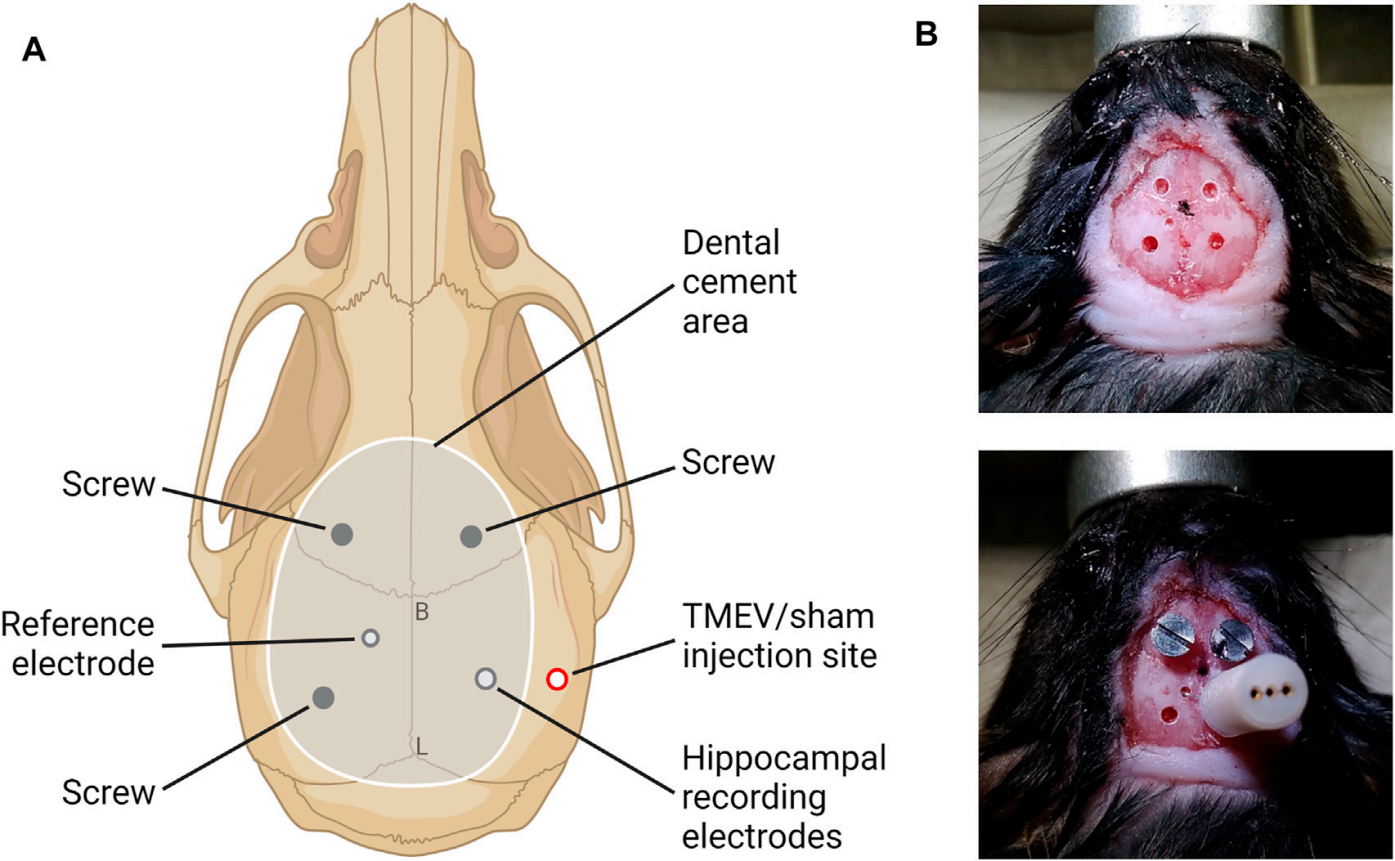
Depiction of the placement of electrodes and the electrodes-anchoring assembly in mice for ECoG. **(A)** Schematic of the mouse skull showing the location for electrodes and anchoring screws over the dorsal surface of skull (B–bregma, L–lambda). This schematic was created with BioRender.com. **(B)** Photographs of the mouse head captured during surgical procedure showing the holes drilled in skull (upper panel) and the implant of electrodes and anterior screws (lower panel).

Mice were anesthetized using 3% isoflurane in oxygen, provided analgesia (0.1 mg/kg buprenorphine intraperitoneal (i.p). and 5 mg/kg carprofen (i.p.), and the head was affixed into a stereotaxic instrument (David Kopf Instruments). The hair over the skull area was removed using a hair-removal cream, the surgical area was cleaned and disinfected using iodine and 70% alcohol, and the skull was exposed. The pitch of head was adjusted to flatten the skull surface by setting the dorsoventral levels of bregma and lambda the same. Mice were continuously anesthetized using nasal tubing supplying 1–2% isoflurane throughout the surgical procedure. Total five holes (three for screws and two for electrodes) were drilled in the skull carefully without causing bleeding (Figure 1). Two electrodes of a three-channel electrode set up (MS333/8-A, P1 Technologies) were twisted and implanted in the CA3 region of hippocampus using stereotaxic coordinates of 1.8 mm lateral (ipsilateral to the infection site) and 2.00 mm posterior from bregma, and about 2.5 mm ventral from the skull surface to reach the target area at 1.8 mm ventral from the brain surface. The reference electrode was implanted in the contralateral cortical surface (1.0 mm lateral and 1.0 mm posterior from bregma). Two anchor screws were placed in the skull bilaterally anterior to the bregma, and the third screw was anchored over the left parietal cortex posterior to the reference electrode. The screws were carefully inserted into the skull without damaging the brain surface. The electrodes and all the screws were secured in position using a dental cement and the skin incision was closed using a tissue glue such that it provided a clear unobstructed space on the right hemisphere for TMEV/sham i. c. injection later (Figure 1). All surgically operated mice were treated humanely and provided post-operative care as per the NIH guidelines and the institutional animal care protocol.

### vECoG and Seizure Analysis

After 10 days of recovery from the surgical procedure, mice were then enrolled for vECoG recording by connecting to an EEG100C differential amplifier (BIOPAC Systems) using a lightweight three-channel cable with a three-channel rotating commutator (P1 Technologies). The MP160 data acquisition system and AcqKnowledge 5.0 software from BIOPAC Systems, Inc. were used to record ECoG. M1065-L network camera (Axis communications) and media Recorder 4.0 software (Noldus Information Technology) were used to record the behavior of each mouse. All the cables and electrical components were sufficiently shielded to minimize electrical noise. Video and EEG recordings were automatically synchronized using the Observer XT 14.1 software (Noldus Information Technology). ECoG signals were bandpass filtered between 0.5 and 100 Hz, amplified, and digited at a sampling frequency of 500 Hz. Mice had access to food and water conveniently during the entire vECoG recording. Mice were injected with 20 µl of TMEV (3.5 × 10^5^ PFU) or PBS in the right somatosensory cortex as described above 1 day after the beginning of recording to get a 24-h baseline recording, and then the recording was continuously acquired for 100 days post-infection. The ECoG and video recordings were reviewed manually by an experimenter blinded to the treatment groups. Electrographic seizures were defined as rhythmic spikes or sharp-wave discharges with amplitudes at least two times the average amplitude of baseline, frequency at least 2 Hz, and duration at least 5 s. Seizures were also identified by verifying postictal suppression of the baseline ECoG activity which typically occurs after seizure but not accompanied by electrographic artifacts associated with mouse behavior other than seizures. At the end of the experiment, mice were transcardially perfused with ice-cold PBS followed by 4% paraformaldehyde, and the brains were collected, and the placement of the electrode was verified.

### Statistics

Datasets with continuous variables are summarized by plotting mean and the standard error of mean as a scatter plot with bar showing individual datapoints, and the datasets with ordinal variables are represented by frequency distribution unless otherwise stated. Parametric statistical tests were used if the data were sufficiently normal distributed and variance within groups was sufficiently similar. Experimental designs with two comparison groups were analyzed by a two-tailed unpaired *t*-test and designs with more than two comparison groups were analyzed using either one-way or two-way analysis of variance (ANOVA). Multiple pairwise comparisons were performed by Tukey’s or Šídák’s posttest. Average cumulative seizure burden was calculated from a ranked dataset and analyzed by Scheirer–Ray–Hare test, which is an extension of the Kruskal–Wallis test for two randomized factorial designs (Scheirer et al., 1976). Two groups with binomial outcome were analyzed by Fisher’s exact test. Survival (percent seizure free) curves were analyzed by log-rank test. The difference between the two groups was considered statistically significant with a *p*-value less than 0.05. GraphPad Prism 8 and Microsoft Excel software were used for statistical analysis.

## Results

### Positive Correlation Between TMEV-Induced Seizures and mBDNF Level in Hippocampus

Transcription of the Bdnf gene can yield three distinct *BDNF*-derived bioactive proteins, namely proBDNF, mature BDNF (mBDNF), and the prodomain of BDNF (Sasi et al., 2017). Opposing effects of proBDNF and mBDNF on neuronal excitability and synaptic strength have been demonstrated through their preferential binding to p75 neurotrophin receptor (p75NTR) and TrkB receptor, respectively. Overall, the mBDNF-TrkB signaling contributes to neuronal hyperexcitability, whereas the proBDNF-p75NTR signaling dampens it (Sasi et al., 2017). To investigate any correlation between the level of mBDNF during the development of TMEV-induced seizures, we measured the level of mBDNF in the hippocampus of TMEV-infected mice by ELISA at various stages post-infection. The timepoints chosen were 1 dpi (before appearance of acute seizures), 3 dpi (beginning of acute seizures), 5 dpi (peak level of acute seizures), 14 dpi (latent period with no behavioral seizure) and 60 dpi (chronic epilepsy period with the occurrence of spontaneous seizures). Since previous studies had found differences in inflammatory and redox markers between ipsilateral and contralateral hippocampus after TMEV infection (Bhuyan et al., 2015), the level of mBDNF was measured in ipsilateral and contralateral hippocampus distinctly.

**FIGURE 2:**
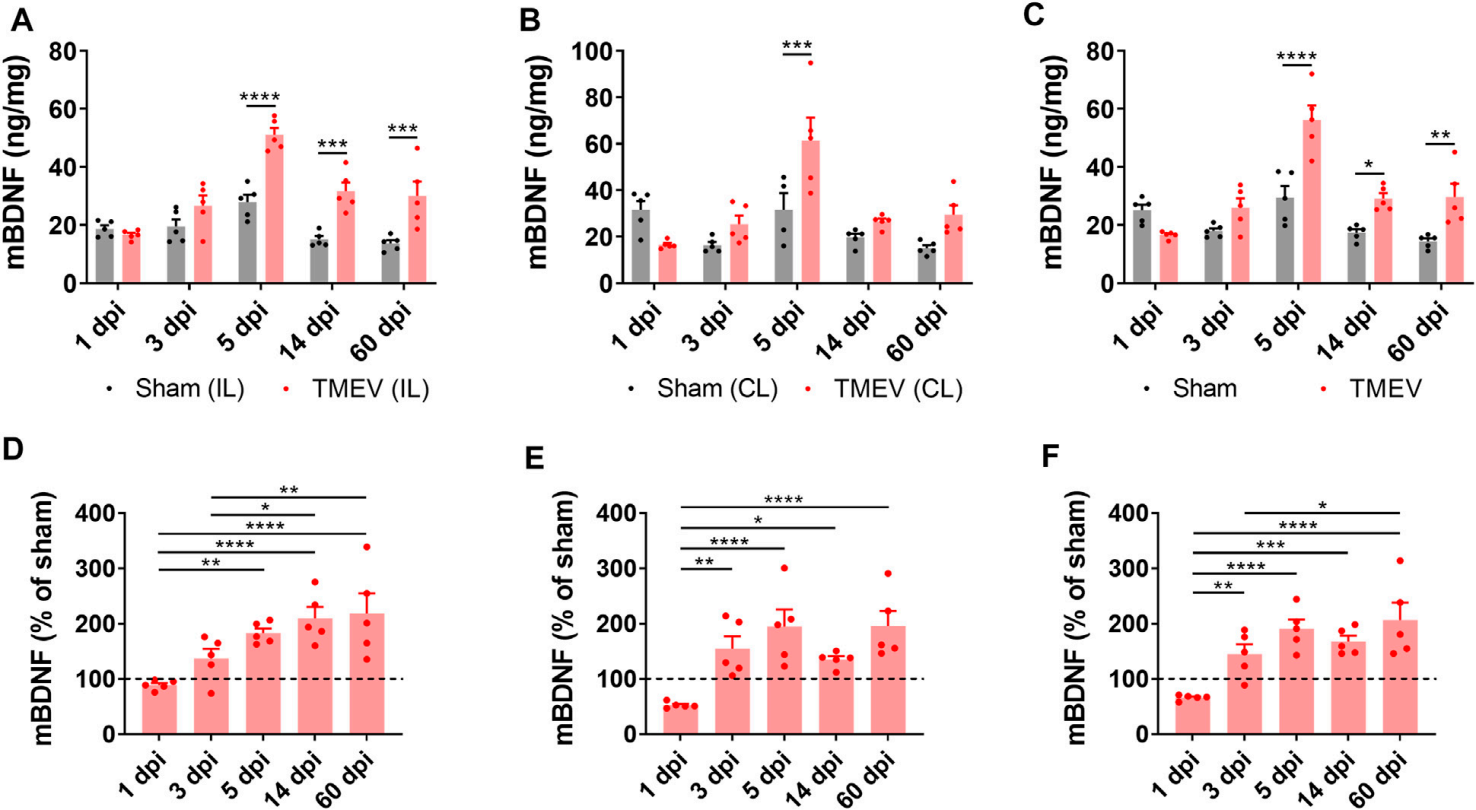
Increase in the protein level of mature BDNF in the hippocampus of TMEV-infected mice during acute and chronic seizure period. **(A-C)** Absolute protein levels of mBDNF in the ipsilateral **(A)**, contralateral **(B)**, and whole hippocampus **(C)** are plotted at various days post-infection (dpi). **(D-F)** The protein level of mBDNF in the ipsilateral **(D)**, contralateral **(E)**, and whole hippocampus **(F)** at various timepoints after infection are normalized to their corresponding levels in the sham-treated control samples. Dashed line at 100% on *y*-axis indicates mean level of mBDNF in the sham-treated control samples. Statistics: Two-way ANOVA, Šídák’s multiple comparison tests (comparison between the treatment groups at each timepoint, panels **A–C**) and Tukey’s multiple comparisons tests (comparison between various timepoints within each treatment group, panels D–F); *n* = 5 mice per group at each timepoint; *p < 0.05, **p < 0.01, ***p < 0.001, ****p < 0.0001.

Figure 2 shows the absolute level of mBDNF in ipsilateral (panel A), contralateral (panel B), and in whole hippocampus (panel C). All the mice in the TMEV-infected group had seizures, whereas none of the sham-treated mice experienced any seizure. The level of mBDNF was not different between both the treatment groups at 1 dpi in both hippocampi. mBDNF started to increase with the appearance of seizures at 3 dpi and was significantly increased compared to the control mice at 5 dpi. The absolute level of mBDNF remained significantly elevated at 14 and 60 dpi in ipsilateral and in whole hippocampus. The changes in the mBDNF level in the infected mice can be conveniently viewed as a percentage of the corresponding control levels in panels D–F. These results show a positive correlation between the occurrence of TMEV-induced seizures and an increase in the levels of mBDNF in hippocampus. The level of mBDNF in the hippocampus of sham-injected control mice did not change significantly over 60 dpi. Since mice in this experiment were not monitored continuously for the seizures by vECoG, it was not possible to determine which mice had chronic seizures at 60 dpi. Although TMEV-infected mice generally do not show behavioral seizures between 2 weeks and 2 months post-infection, focal electrographic seizures have been recorded in the hippocampus during this period (Patel et al., 2017). It is likely that increased level of mBDNF in hippocampus at 60 dpi could be due to underlying network hyperexcitability. However, further work is needed to determine whether increase in mBDNF found in hippocampus at 60 dpi is mainly due to occurrence of chronic seizures or due to other pathological factors.

### Increase in the Ratio of NKCC1/KCC2 in Hippocampus During Acute TMEV Infection

**FIGURE 3:**
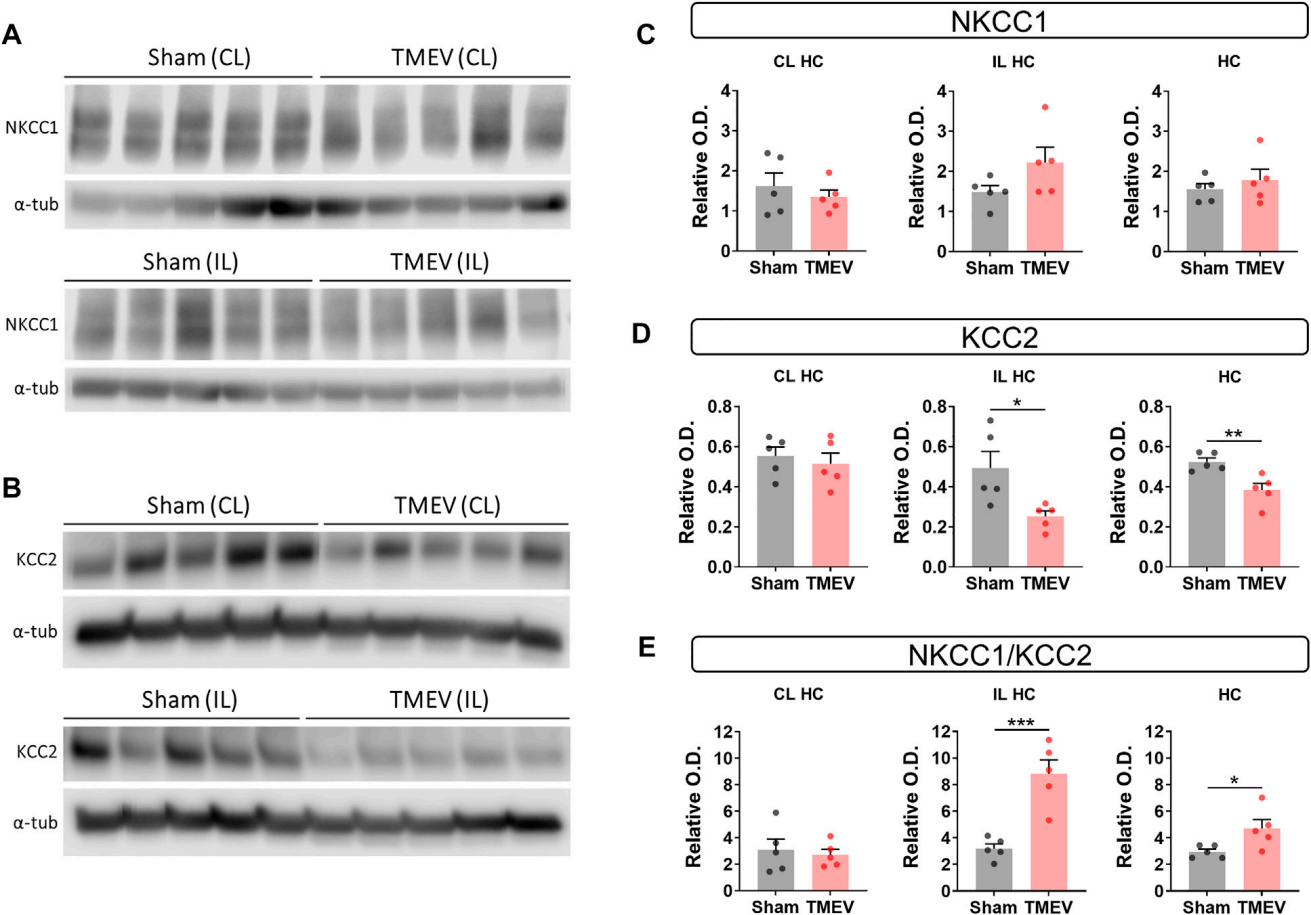
Decrease in the protein level of KCC2 and increase in the ratio of NKCC1/KCC2 in the hippocampus of TMEV-infected mice with acute seizures at 5 dpi. **(A–B)** Immunoblots show the detection of NKCC1 **(A)**, KCC2 **(B)** and α-tubulin in the contralateral (CL) and ipsilateral (IL) hippocampi (HC) from the sham- and TMEV-infected mice. **(C–E)** Optical density (O.D.) of NKCC1 **(C)** and KCC2 **(D)** blots were normalized to the O.D. of α-tubulin blots from the same samples to compare the relative O.D. between the sham- and TMEV-infected mice. The relative O.D. ratios of NKCC1/KCC2 are plotted in panel **(E)**. Statistics: Unpaired two-tailed *t* test; *n* = 5 mice per group; *p < 0.05, **p < 0.01, ***p < 0.001.

Whether increased levels of mBDNF are associated with the changes in the expression of cation-chloride cotransporters, we measured the level of NKCC1 and KCC2 in ipsilateral and contralateral hippocampus by western blot at 5 dpi. We found no change in NKCC1 in both hippocampi (Figures 3A,C), whereas KCC2 was found significanlty reduced in ipsilaeral hippampus and unchanged in contralateral hippocampus (Figures 3B,D). Overall the level of KCC2 in whole hippocampus was significanlty reduced (Figure 3D). Accordingly, the ratio of NKCC1/KCC2 was significantly elevated in the ipsilateral hippocampus (Figure 3E).

### Neutralization of BDNF Rescues Reduction in Hippocampal KCC2 in Mice with TMEV-Induced Seizures

**FIGURE 4:**
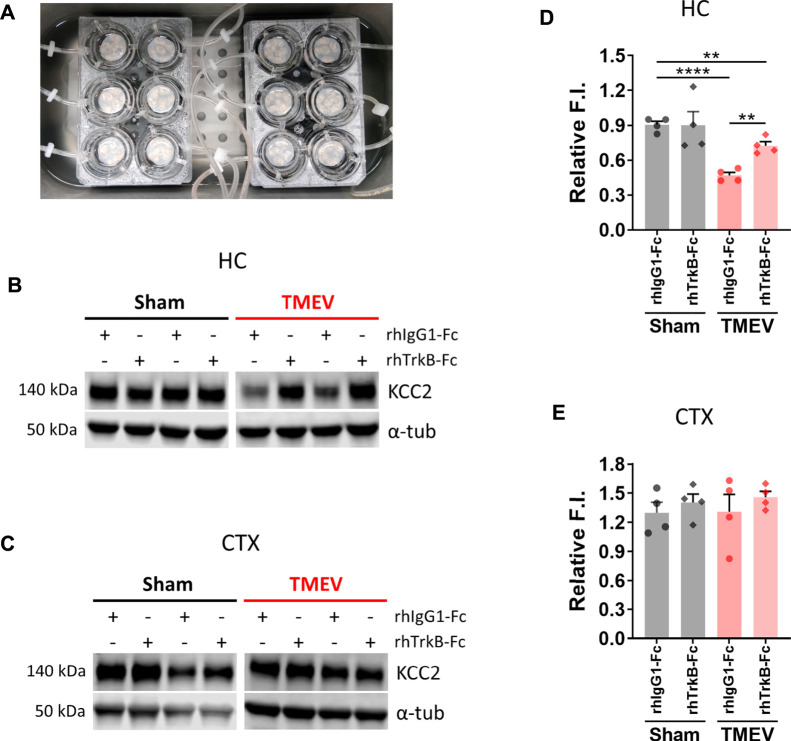
Inhibition of BDNF increases the expression of KCC2 in hippocampal slices from mice during TMEV-induced acute seizure period. **(A)** Acute brain slices (300 µm thickness) from sham- or TMEV-infected mice were treated with rhTrkB-Fc or rhIgG1-Fc (250 ng/ml in ACSF) for 4.5 h at 37 C under continuous carbogenation (95% oxygen, 5% carbon dioxide) condition before dissecting out hippocampus (HC) and cortex (CTX). **(B–C)** Representative immunoblots show the detection of KCC2 and α-tubulin in hippocampi **(B)** and cortex **(C)** isolated from acute brain slices under different treatment conditions for both groups of mice. **(D–E)** Fluor intensity (F.I.) of KCC2 blots were normalized to the F.I. of α-tubulin blots from the same samples to compare the relative F.I. between the sham- and TMEV-infected slices treated either with rhIgG1-Fc or rhTrkB-Fc. Statistics: One-way ANOVA, Tukey’s multiple comparisons test; *n* = 4 mice per group; **p < 0.01, ****p < 0.0001.

We next investigated any causality between an increase in mBDNF and a concurrent decrease in KCC2 in the hippocampus during peak TMEV-induced acute seizures using a recombinant human TrkB-Fc (rhTrkB-Fc), a biological inhibitor of BDNF signaling, in acute brain slices using western blot. rhTrkB-Fc is a decoy scavenging body that binds and sequesters BDNF, and thus inhibits the downstream effects of BDNF. Intracerebroventricular administration of rhTrkB-Fc inhibited the development of seizures in the rat model of electrical kindling-induced epilepsy (Binder et al., 1999). However, a major disadvantage of *in vivo* delivery of rhTrkB-Fc is a poor parenchymal penetration, and therefore, limited bioavailability of rhTrkB-Fc at the target site (Binder et al., 1999). To overcome this limitation, we incubated acute brain slices prepared at 5 days post-TMEV/sham treatment with rhTrkB-Fc or rhIgG1-Fc at 37°C in a custom-built six well incubation plate designed to provide continuous supply of 95% oxygen and 5% carbon dioxide (Figure 4A). The slices remained viable and healthy throughout the process using this system. The concentration of rhTrkB-Fc or rhIgG1-Fc (250 ng/ml) and incubation duration (4.5 h) were based on previously published studies and our pilot experiments (Rivera et al., 2002).

The results showed that scavenging endogenous BDNF significantly increased KCC2 level in the infected hippocampal slices (Figures 4B,D). However, it did not completely reverse the reduction in KCC2 as there was a small but significant difference between the sham and TMEV groups. We also measured the effect of rhTrkB-Fc on KCC2 in cortical slices, where we do not find a reduction in KCC2 during TMEV-induced seizures. There was no change in the level of KCC2 between any of the treatment groups–cortical slices from sham or TMEV-infected mice treated with rhTrkB-Fc or rhIgG1-Fc (Figures 4C,E). These results suggest that increased release of endogenous BDNF during TMEV-induced acute seizures directly decrease the expression of KCC2, which in turn, may contribute to hyperexcitability by impairing GABAergic inhibition.

### BDNF Heterozygous Knockout and Wild-type C57BL/6J Mice Are Equally Susceptible to TMEV-Induced Seizures

**FIGURE 5:**
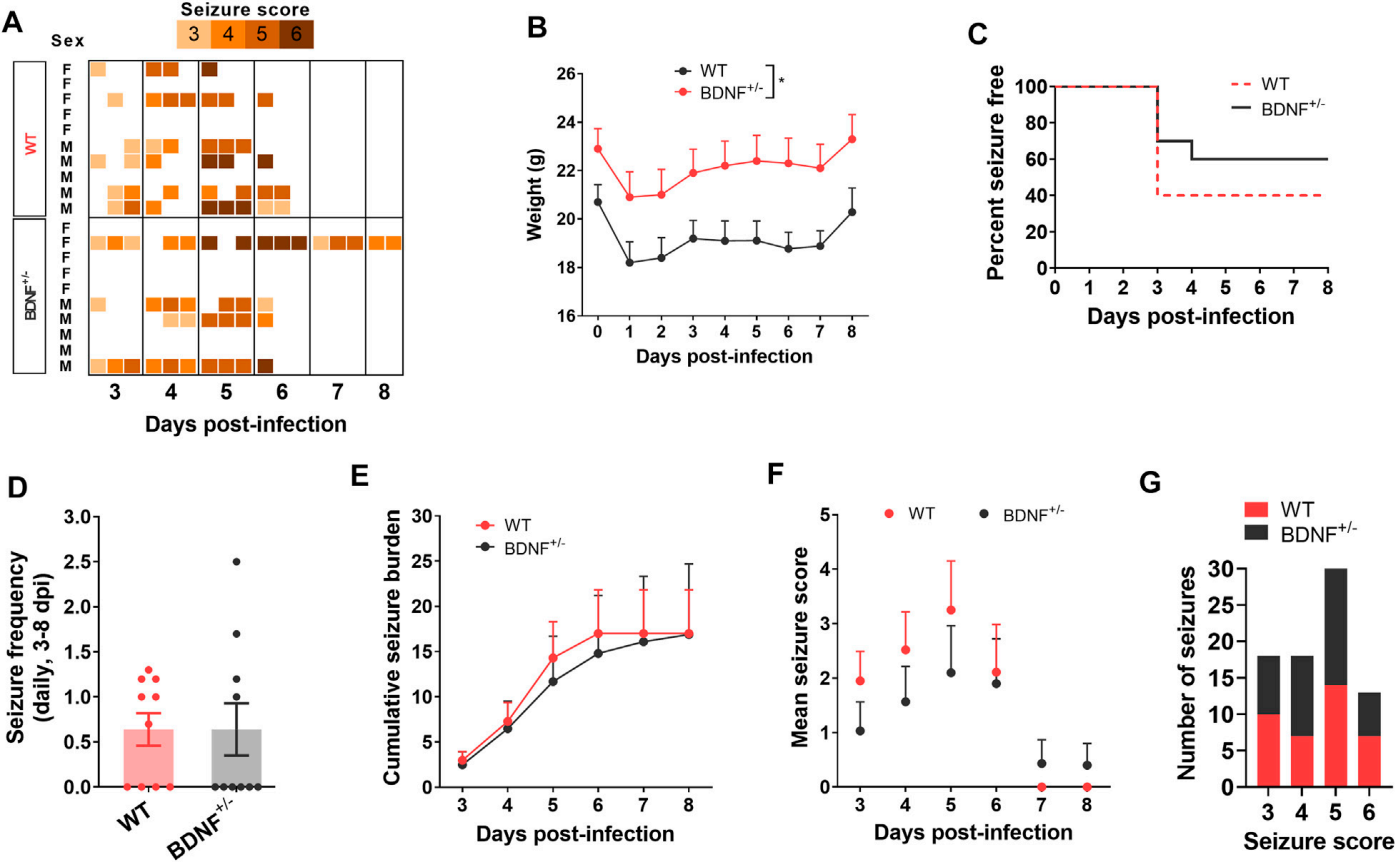
No difference in TMEV-induced acute seizures between wild type (WT) and BDNF^+/−^ C57BL/6J mice. **(A)** Heatmap shows all acute seizures observed based on their severity score for each mouse between 3–8 days post-infection (dpi). Seizures were induced by handing and the severity was scored three times a day with at least 2 h of undisturbed period between two observation sessions. **(B)** Average weight of mice in each group during acute infection period. **(C)** This panel shows the percentage of total infected mice in each group that remained seizure free at each day until 8 dpi. None of the mice developed seizures before 3 dpi. **(D)** Average number of seizures daily between 3-8 dpi is plotted for each mouse. **(E)** Average cumulative seizure burden, which is calculated as a mean of the summation of all seizure scores for each mouse over time, compares seizure severity between WT and BDNF^+/−^ mice during acute infection period. **(F)** Mean seizure score at each day is compared. **(G)** It shows the total number of seizures based on their severity score.

To test whether enhanced level of BDNF directly contributes to seizures, we compared the seizure susceptibility of BDNF^+/−^ mice to TMEV-induced seizures with the WT C57BL/6J mice. It was desirable to utilize mice lacking both alleles of the *Bdnf* gene; however, *BDNF* ^−/−^ mice are growth-impaired and most die within 2 weeks of birth, whereas BDNF^+/−^ mice are phenotypically comparable with the WT mice (Ernfors et al., 1994). Both BDNF^+/−^ and WT mice (*n* = 10 per group; 5 male, 5 female) were infected with TMEV and monitored for behavioral seizures up to 2 weeks post-infection by an experimenter blinded to the genotype of mice. All the seizures observed are shown in the heatmap in Figure 5A. Average weight of BDNF^+/−^ mice was about 2–3 g more than the WT mice (Figure 5B). Although 40% (4/10) of BDNF^+/−^ mice developed acute seizures compared to 60% (6/10) of WT mice, this difference was not statistically significant (Figure 5C). Mean seizure frequency was measured as an average number of seizures during the entire acute seizure period (3-8 dpi) and found similar between both the groups (Figure 5D). Cumulative seizure burden, as a marker for seizure severity, at each dpi for each mouse was calculated by summing all its seizure scores up to corresponding dpi. Seizure severity was also found similar between both the groups (Figure 5E). Comparisons based on seizure score found no difference between both the groups (Figures 5F,G).

**FIGURE 6:**
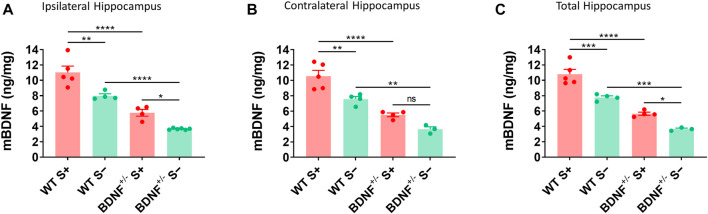
Increase in mBDNF in hippocampus of WT compared to BDNF^+/−^ mice with TMEV-infected acute seizures. **(A-C)** Absolute protein levels of mBDNF in the ipsilateral **(A)**, contralateral **(B)**, and total hippocampus **(C)** from WT and BDNF^+/−^ mice at 14 days post-TMEV infection are compared. S+ indicates mice that developed acute TMEV-induced seizures and S- indicates mice that did not get seizures. Statistics: One-way ANOVA, Šídák’s multiple comparisons test; *n* = 3-6 mice per group at each timepoint; *p < 0.05, **p < 0.01, ***p < 0.001, ****p < 0.0001.

The level of mBDNF was measured by ELISA in ipsilateral (Figure 6A), contralateral (Figure 6B), and whole (Figure 6C) hippocampus collected from BDNF^+/−^ and WT mice at 14 dpi. BDNF^+/−^ mice had about half the level of mBDNF compared to the WT mice irrespective of seizures. Mice that exhibited TMEV-induced acute seizures had significantly higher level of BDNF irrespective of their genotype compared to mice protected from acute seizures. These results suggest that upregulation of endogenous BDNF in hippocampus occurs as a consequence of acute seizures rather than TMEV infection itself.

### No Effect of pY816-Mediated Inhibition of the BDNF-TrkB-PLCγ1 Signaling on TMEV Infection-Associated Seizures

To overcome the limitations of inhibiting endogenous release of BDNF, we employed a pharmacological approach using a peptide, pY816, to inhibit the downstream effects of BDNF mediated through TrkB receptor. Among the many downstream effector molecules that mediate the effects of BDNF through TrkB receptors, activation of PLCγ1 has been implicated in driving seizures (Gu et al., 2015). pY816 is a selective inhibitor of the PLCγ1-mediated effects of the BDNF-TrkB signaling. It is a membrane-permeable peptide containing a sequence of TrkB motif that directly binds with PLCγ1 and uncouples the interaction of PLCγ1 with activated TrkB, and thus, inhibits the downstream effects of BDNF (Gu et al., 2015). pY816 has been found to reduce seizures and epileptogenesis in the rodent models of seizures induced by kainic acid (Gu et al., 2015) or electrical kindling (Krishnamurthy et al., 2019). Since the BDNF-TrkB-PLCγ1 signaling cascade, along with the Shc/FRS-2 (src homology 2 domain containing transforming protein/FGF receptor substrate 2) pathway, was also implicated in downregulating KCC2 expression in hippocampal slices under hyperexcitable condition (Rivera et al., 2004), we asked whether treatment of TMEV-infected mice with pY816 might reduce acute and/or chronic seizure parameters.

**FIGURE 7:**
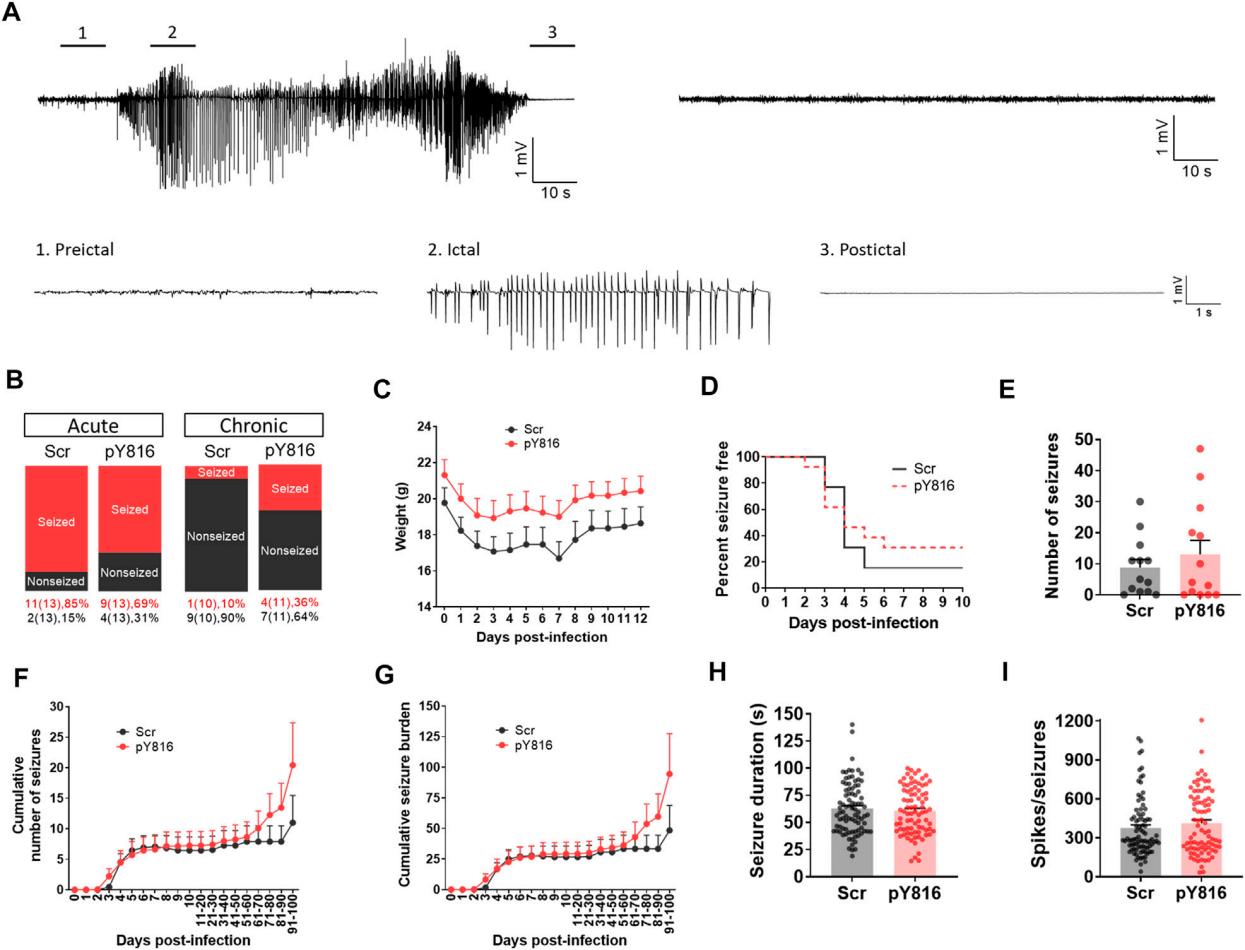
No change in frequency, severity, and duration of TMEV-induced acute and chronic seizures between pY816- and sham-treated mice. **(A)** Representative EEG recordings from TMEV-infected mice treated with a scrambled control peptide (Scr). The recording on the upper left is from a mouse that developed seizures and the recording on the upper right is from a mouse that did not have any seizure. Expanded traces corresponding to preictal baseline, ictal spiking, and postictal suppression period from a tracing on upper left are shown below. **(B)** Percentage of all TMEV-infected mice treated with either pY816 or a scrambled control peptide (Scr) that presented with acute and chronic seizures are shown. **(C)** Average weight of mice in each group during acute infection period. **(D)** This panel shows the percentage of total infected mice in each group that remained seizure free at each day until 10 dpi. None of the mice developed seizures before 2 dpi. **(E)** Total number of seizures throughout the vECoG monitoring period post-TMEV infection is plotted for each mouse. **(F)** Average cumulative number of seizures for each treatment group over 100 days of vECoG monitoring post-TMEV infection. **(G)** Average cumulative seizure burden, which is calculated as a mean of the summation of all seizure scores for each mouse over time, compares seizure severity between Scr-treated and pY816-treated mice up to 100 dpi. **(H)** A graph plotting the duration of all seizures show no difference in mean seizure duration between both the treatment groups. **(I)** Total number of spikes plotted for each electrographic seizure shows no mean difference between both the treatment groups.

We treated TMEV-infected mice with pY816 or Scr (*n* = 13 per group) once daily during the first 2 weeks of infection and monitored them for seizures by continuous vECoG until 100 dpi. The recoding from three mice in the Scr group and from two in the pY816 group had to be stopped before 100 dpi due to loss of electrode or poor health. The representative recording traces from the Scr-treated mice with and without seizures are shown in Figure 7A. The recording from the pY816-treated mice also showed similar electrographic activity. Numbers of mice that had at least one acute seizure were similar for both the groups (Scr–11/13, 85%; pY816–9/13, 69%) (Figure 7B). Numbers of mice with chronic spontaneous seizures during 60–100 dpi were higher in the pY816 group (Scr–1/10, 10%; pY816–4/11, 36%); however, this difference was not statistically significant (Figure 7B). There was no difference in weight, occurrence of first seizure, and average number of seizures between both the groups (Figures 7C–E). Seizure frequency and severity, reported as cumulative number of seizures and cumulative seizure burden, respectively, were also not different between both the groups (Figures 7F,G). Seizure duration and number of spikes per ictal activity were calculated from the vECoG recording and found similar for both the treatment groups (Figures 7H,I).

## Discussion

We have investigated the role of the BDNF-TrkB signaling in the development of seizures in a model of TMEV infection-associated limbic epilepsy. We found an inverse correlation between the protein levels of BDNF and KCC2 in mice hippocampus during the peak seizure activity period following viral infection. Increased expression of BDNF was found coincident with the occurrence of both acute and chronic seizures. We also found a causal connection between the level of BDNF and KCC2 as neutralizing endogenous BDNF by rhTrkB-Fc significantly increased the level of KCC2 in hippocampal slices from the mice with acute seizures. However, genetic and pharmacological approaches to inhibit excessive effects of BDNF did not show any difference in behavioral and electrographic seizures induced by TMEV. Overall, our results suggest that increased release of BDNF may contribute to TMEV infection-induced seizures by impairing KCC2-mediated regulation of [Cl^−^]_i_ important for maintaining fast GABAergic inhibition.

The role of BDNF in seizure development has been reported in some animal models of seizures and epilepsy, mostly electrical kindling, and post-status epilepticus (SE) models of TLE using kainic acid and pilocarpine (McNamara and Scharfman, 2012). These are useful and reliable methods to induce seizures, however, they are limited by the fact that normal healthy rodents are used in these models to induce seizures and that *de novo* SE is rather rare in humans and thus not a major cause of acquired epilepsy (Loscher, 2017). Animal models of acquired epilepsy caused by stroke, CNS infections, or traumatic brain injury, which are the major etiological factors for human acquired epilepsy, are more clinically relevant. Viral infection is a major cause of TLE especially in LMIC (Misra et al., 2008; Vezzani et al., 2016). However, animal models of TLE caused by infection are limited. Furthermore, high mortality rate and low rate of epilepsy development in majority of these models curtail their utility (Patel and Wilcox, 2017; Löscher and Howe, 2022). In contrast, TMEV-infected C57BL/6J mice exhibit acute seizures during active infection, survive the initial infection, exhibit hippocampal neuropathology and gliotic scar, develop behavioral comorbidities such as anxiety and cognitive impairment, and develop epilepsy after about 2–3 months post-infection (Libbey et al., 2008; Stewart et al., 2010a; b;Umpierre et al., 2014; Broer et al., 2016; Patel et al., 2017; Lawley et al., 2021). This model recapitulates many pathological and behavioral sequelae of human TLE patients, and therefore, offers a unique opportunity to study the molecular mechanism(s) underlying seizure generation and epileptogenesis.

The factors that upregulate BDNF expression during TMEV-induced seizures are speculative. Inflammation is a key driver of seizures in the TMEV model (Cusick et al., 2013; Patel et al., 2017; Waltl et al., 2018; Zhan et al., 2018; Juda et al., 2019; Howe et al., 2022). Similar to BDNF, increase in the levels of proinflammatory cytokines and chemokines correlates with the appearance and persistence of TMEV-induced seizures. Persistent inflammatory condition has been shown to upregulate BDNF level (Tao et al., 2014). Reactive microgliosis, which occurs during acute TMEV infection and known to release proinflammatory factors, can induce BDNF synthesis and release through the ATP-P2X4R signaling cascade (Ferrini and De Koninck, 2013). However, the interaction between BDNF and neuroinflammation in CNS disorders is not clearly understood as evident by many contrasting findings (Lima Giacobbo et al., 2019). Ca^2+^-dependent synaptic release of BDNF has been demonstrated in response to spontaneous activity (Kuczewski et al., 2008), therefore, it is also likely that the network hyperexcitability ensued following TMEV infection could itself have contributed to the increased levels of BDNF. Since BDNF can cause hyperexcitability through intracellular Ca^2+^-dependent mechanisms (Sasi et al., 2017), it is possible that there may be a positive feedback loop between the release of BDNF and seizures.

Accumulating evidence suggest that BDNF decreases KCC2 expression and function in several adult brain regions (Moore et al., 2017), under different neuropathological conditions including epilepsy (Rivera et al., 2002), traumatic brain injury (Shulga et al., 2008), and nociception (Ferrini et al., 2013). In a mature brain, cell surface expression of KCC2 is crucial for keeping [Cl^−^]_i_ low, which is essential for establishing and maintaining fast hyperpolarizing GABA_A_ receptor currents. A decrease in the Cl^−^ extrusion function of KCC2 causes depolarizing excitatory shift in the GABAergic action which may underlie neuronal network hyperexcitability. Impairment in the function of KCC2 has been reported in many experimental studies from the animal models of epilepsy as well as in some cases of human epilepsy (Moore et al., 2017). Although the consequence of reduced KCC2 level on the fast GABA_A_ receptor inhibition following TMEV infection is not studied in this present study, an impaired synaptic inhibition during both acute and chronic phases of TMEV-induced epilepsy has been reported in the CA3 region of hippocampus (Smeal et al., 2015) and in the dentate granule cells (our unpublished data). It is likely that BDNF-mediated reduction in KCC2 function may have contributed to this impaired inhibition.

In addition to BDNF, high extracellular glutamate has been shown to reduce KCC2 level through NMDA receptor and subsequently calcium-activated protease (Lee et al., 2011). Increased extracellular glutamate is commonly associated with neuronal network hyperexcitability (Coulter and Steinhauser, 2015; Patel et al., 2019a). Glutamate-mediated hyperexcitability occurs in CA3 pyramidal neurons during acute and chronic seizure periods in the TMEV model of epilepsy (Smeal et al., 2012). Given these findings, it is possible that excessive glutamate may have reduced KCC2 in hippocampus during acute TMEV-induced seizures. However, we have demonstrated a significant increase in KCC2 in TMEV-infected hippocampal slices after treatment with BDNF scavenging bodies. Although the level of KCC2 was still lower compared to control hippocampal slices, the reduction was much less compared to TMEV-infected hippocampal slices without any alternation of endogenous BDNF. Collectively, these results suggest that BDNF directly contributes in the reduction of KCC2 during TMEV-induced seizures, however, it may not be the only factor.

Our attempts to inhibit the consequences of increased release of BDNF either through genetic or pharmacological approaches did not affect acute as well as chronic seizures caused by TMEV infection. In contrast to the TMEV model, deletion of single allele of BDNF gene was sufficient to inhibit the development of kindling in mice (Kokaia et al., 1995). As expected, the level of BDNF in the hippocampus of BDNF^+/−^ mice was at half the level found in the WT mice. TMEV-infected BDNF^+/−^ mice that did not have seizures had much less amount of BDNF in hippocampus compared to BDNF^+/−^ mice that had seizures affirming a positive correlation between seizures and BDNF level. Alternatively, pharmacological inhibition of TrkB receptor has been attempted in other epilepsy models (Lin et al., 2020). Since TrkB also mediates neuroprotective effects of BDNF and the PLCγ1-mediated effects of the BDNF-TrkB signaling was primarily identified to impart antiseizure effects, pY816 was rationally designed to uncouple PLCγ1 from activated TrkB (Gu et al., 2015). Treatment of mice with pY816 (10 mg/kg, i. v.) once daily for 3 days following kainic acid-induced SE not only inhibited seizures within first 2 weeks post-SE but also prevented the development to epilepsy 5–6 weeks post-SE (Gu et al., 2015). Our pilot studies informed that this dosing regimen of pY816 may not be enough to control TMEV-induced seizures. Therefore, we treated TMEV-infected mice with pY816 (10 mg/kg, i. v.) once daily for the first 2 weeks starting immediately after infection as TMEV is cleared from the brain after 2 weeks of infection (Libbey et al., 2010). We chose retroorbital route for i. v. dosing because it is effective and less stressful method for chronic i. v. treatment (Steel et al., 2008). However, increasing the duration of pY816 treatment did not affect TMEV-induced seizures. In contrast to kindling and SE models of epilepsy, lack of effects on TMEV-induced seizures observed in pY816-treated and in BDNF^+/−^ mice likely reflects model-specific differences in the mechanisms of ictogenesis. It is possible that the absence of anticonvulsant effects of pY816 observed in TMEV-infected mice could be due to lack of its effects on KCC2. However, further studies are needed to measure the level of KCC2 during the chronic TMEV infection and at various timepoints following pY816 treatment. Furthermore, BDNF-mediated downregulation of KCC2 required activation of both PLCγ1-CREB (phospholipase Cγ1–cAMP response element-binding protein) and Shc/FRC-2 (src homology 2 domain containing transforming protein/FGF receptor substrate 2)-mediated signaling cascade (Rivera et al., 2004). In fact, BDNF either had no effect on or increased the level of KCC2 respectively in mice with mutation in TrkB receptor that permitted the downstream signaling specifically through the PLCγ1-CREB or ShC/FRC-2 pathway (Rivera et al., 2004). It is likely based on this finding that uncoupling PLCγ1 from the activated TrkB alone using pY816 could be insufficient to ameliorate the reduction of KCC2 and suppress TMEV-induced seizures. Lastly, since excessive inflammatory reaction post-TMEV infection is a major driver for the development of seizures in this model (Löscher and Howe, 2022), it is likely that inhibiting BDNF signaling mediated through PLCγ1 alone may not have significant anticonvulsant effects.

In conclusion, the present work suggests that increased release of BDNF could contribute to the development seizures and epilepsy induced by TMEV infection. BDNF could cause neuronal network hyperexcitability by reducing the expression of KCC2, thereby impairing KCC2-mediated chloride homeostasis critical for maintaining inhibitory control over neural network. Further studies are warranted to test the strategies for modulating the BDNF-TrkB signaling and their impact on chloride homeostasis and occurrence of infection-associated seizures.

## Data Availability

The original contributions presented in the study are included in the article/supplementary material, further inquiries can be directed to the corresponding author.

## References

[B1] Beghi E. (2020). The Epidemiology of Epilepsy. Neuroepidemiology 54, 185–191. 10.1159/000503831 31852003

[B2] Bhuyan P. Patel D. C. Wilcox K. S. Patel M. (2015). Oxidative Stress in Murine Theiler's Virus-Induced Temporal Lobe Epilepsy. Exp. Neurol. 271, 329–334. 10.1016/j.expneurol.2015.06.012 26079647 PMC4848026

[B3] Binder D. K. Routbort M. J. Ryan T. E. Yancopoulos G. D. Mcnamara J. O. (1999). Selective Inhibition of Kindling Development by Intraventricular Administration of TrkB Receptor Body. J. Neurosci. 19, 1424–1436. 10.1523/jneurosci.19-04-01424.1999 9952419 PMC6786043

[B4] Bröer S. Käufer C. Haist V. Li L. Gerhauser I. Anjum M. (2016). Brain Inflammation, Neurodegeneration and Seizure Development Following Picornavirus Infection Markedly Differ Among Virus and Mouse Strains and Substrains. Exp. Neurol. 279, 57–74. 10.1016/j.expneurol.2016.02.011 26892877

[B5] Coulter D. A. Steinhauser C. (2015). Role of Astrocytes in Epilepsy. Cold Spring Harb. Perspect. Med. 5, a022434. 10.1101/cshperspect.a022434 25732035 PMC4355248

[B6] Croll S. D. Suri C. Compton D. L. Simmons M. V. Yancopoulos G. D. Lindsay R. M. (1999). Brain-derived Neurotrophic Factor Transgenic Mice Exhibit Passive Avoidance Deficits, Increased Seizure Severity and *In Vitro* Hyperexcitability in the hippocampus and Entorhinal Cortex. Neuroscience 93, 1491–1506. 10.1016/s0306-4522(99)00296-1 10501474 PMC2504500

[B7] Cusick M. F. Libbey J. E. Patel D. C. Doty D. J. Fujinami R. S. (2013). Infiltrating Macrophages Are Key to the Development of Seizures Following Virus Infection. J. Virol. 87, 1849–1860. 10.1128/jvi.02747-12 23236075 PMC3554195

[B8] Doyon N. Vinay L. Prescott S. A. De Koninck Y. (2016). Chloride Regulation: A Dynamic Equilibrium Crucial for Synaptic Inhibition. Neuron 89, 1157–1172. 10.1016/j.neuron.2016.02.030 26985723

[B9] Dugich-Djordjevic M. M. Tocco G. Lapchak P. A. Pasinetti G. M. Najm I. Baudry M. (1992). Regionally Specific and Rapid Increases in Brain-Derived Neurotrophic Factor Messenger RNA in the Adult Rat Brain Following Seizures Induced by Systemic Administration of Kainic Acid. Neuroscience 47, 303–315. 10.1016/0306-4522(92)90246-x 1641125

[B10] Ernfors P. Bengzon J. Kokaia Z. Persson H. Lindvall O. (1991). Increased Levels of Messenger RNAs for Neurotrophic Factors in the Brain during Kindling Epileptogenesis. Neuron 7, 165–176. 10.1016/0896-6273(91)90084-d 1829904

[B11] Ernfors P. Lee K.-F. Jaenisch R. (1994). Mice Lacking Brain-Derived Neurotrophic Factor Develop with Sensory Deficits. Nature 368, 147–150. 10.1038/368147a0 8139657

[B12] Ferrini F. De Koninck Y. (2013). Microglia Control Neuronal Network Excitability via BDNF Signalling. Neural Plast. 2013, 429815. 10.1155/2013/429815 24089642 PMC3780625

[B13] Ferrini F. Trang T. Mattioli T.-A. M. Laffray S. Del'guidice T. Lorenzo L.-E. (2013). Morphine Hyperalgesia Gated through Microglia-Mediated Disruption of Neuronal Cl− Homeostasis. Nat. Neurosci. 16, 183–192. 10.1038/nn.3295 23292683 PMC4974077

[B14] Gu B. Huang Y. Z. He X.-P. Joshi R. B. Jang W. Mcnamara J. O. (2015). A Peptide Uncoupling BDNF Receptor TrkB from Phospholipase Cγ1 Prevents Epilepsy Induced by Status Epilepticus. Neuron 88, 484–491. 10.1016/j.neuron.2015.09.032 26481038 PMC4636438

[B15] He X.-P. Kotloski R. Nef S. Luikart B. W. Parada L. F. Mcnamara J. O. (2004). Conditional Deletion of TrkB but Not BDNF Prevents Epileptogenesis in the Kindling Model. Neuron 43, 31–42. 10.1016/j.neuron.2004.06.019 15233915

[B16] Howe C. L. Lafrance-Corey R. G. Overlee B. L. Johnson R. K. Clarkson B. D. S. Goddery E. N. (2022). Inflammatory Monocytes and Microglia Play Independent Roles in Inflammatory Ictogenesis. J. Neuroinflammation 19, 22. 10.1186/s12974-022-02394-1 35093106 PMC8800194

[B17] Juda M. B. Brooks A. K. Towers A. E. Freund G. G. Mccusker R. H. Steelman A. J. (2019). Indoleamine 2,3‐dioxygenase 1 Deletion Promotes Theiler's Virus-Induced Seizures in C57 BL/6J Mice. Epilepsia 60, 626–635. 10.1111/epi.14675 30770561 PMC8273875

[B18] Kokaia M. Ernfors P. Kokaia Z. Elmér E. Jaenisch R. Lindvall O. (1995). Suppressed Epileptogenesis in BDNF Mutant Mice. Exp. Neurol. 133, 215–224. 10.1006/exnr.1995.1024 7649227

[B19] Kolbeck R. Bartke I. Eberle W. Barde Y. A. (1999). Brain-derived Neurotrophic Factor Levels in the Nervous System of Wild-type and Neurotrophin Gene Mutant Mice. J. Neurochem. 72, 1930–1938. 10.1046/j.1471-4159.1999.0721930.x 10217270

[B20] Krishnamurthy K. Huang Y. Z. Harward S. C. Sharma K. K. Tamayo D. L. Mcnamara J. O. (2019). Regression of Epileptogenesis by Inhibiting Tropomyosin Kinase B Signaling Following a Seizure. Ann. Neurol. 86, 939–950. 10.1002/ana.25602 31525273 PMC7531259

[B21] Kuczewski N. Porcher C. Ferrand N. Fiorentino H. Pellegrino C. Kolarow R. (2008). Backpropagating Action Potentials Trigger Dendritic Release of BDNF during Spontaneous Network Activity. J. Neurosci. 28, 7013–7023. 10.1523/jneurosci.1673-08.2008 18596175 PMC6670985

[B22] Lähteinen S. Pitkänen A. Koponen E. Saarelainen T. Castrén E. (2003). Exacerbated Status Epilepticus and Acute Cell Loss, but No Changes in Epileptogenesis, in Mice with Increased Brain-Derived Neurotrophic Factor Signaling. Neuroscience 122, 1081–1092. 10.1016/j.neuroscience.2003.08.037 14643774

[B23] Lawley K. S. Rech R. R. Elenwa F. Han G. Perez Gomez A. A. Amstalden K. (2021). Host Genetic Diversity Drives Variable Central Nervous System Lesion Distribution in Chronic Phase of Theiler's Murine Encephalomyelitis Virus (TMEV) Infection. PLoS One 16, e0256370. 10.1371/journal.pone.0256370 34415947 PMC8378701

[B24] Lee H. H. C. Deeb T. Z. Walker J. A. Davies P. A. Moss S. J. (2011). NMDA Receptor Activity Downregulates KCC2 Resulting in Depolarizing GABAA Receptor-Mediated Currents. Nat. Neurosci. 14, 736–743. 10.1038/nn.2806 21532577 PMC3102766

[B25] Libbey J. E. Kirkman N. J. Smith M. C. P. Tanaka T. Wilcox K. S. White H. S. (2008). Seizures Following Picornavirus Infection. Epilepsia 49, 1066–1074. 10.1111/j.1528-1167.2008.01535.x 18325012

[B26] Libbey J. E. Kirkman N. J. Wilcox K. S. White H. S. Fujinami R. S. (2010). Role for Complement in the Development of Seizures Following Acute Viral Infection. J. Virol. 84, 6452–6460. 10.1128/jvi.00422-10 20427530 PMC2903264

[B27] Lima Giacobbo B. Doorduin J. Klein H. C. Dierckx R. A. J. O. Bromberg E. De Vries E. F. J. (2019). Brain-Derived Neurotrophic Factor in Brain Disorders: Focus on Neuroinflammation. Mol. Neurobiol. 56, 3295–3312. 10.1007/s12035-018-1283-6 30117106 PMC6476855

[B28] Lin T. W. Harward S. C. Huang Y. Z. Mcnamara J. O. (2020). Targeting BDNF/TrkB Pathways for Preventing or Suppressing Epilepsy. Neuropharmacology 167, 107734. 10.1016/j.neuropharm.2019.107734 31377199 PMC7714524

[B29] Liu G. Gu B. He X.-P. Joshi R. B. Wackerle H. D. Rodriguiz R. M. (2013). Transient Inhibition of TrkB Kinase after Status Epilepticus Prevents Development of Temporal Lobe Epilepsy. Neuron 79, 31–38. 10.1016/j.neuron.2013.04.027 23790754 PMC3744583

[B30] Löscher W. (2017). Animal Models of Seizures and Epilepsy: Past, Present, and Future Role for the Discovery of Antiseizure Drugs. Neurochem. Res. 42, 1873–1888. 10.1007/s11064-017-2222-z 28290134

[B31] Löscher W. Howe C. L. (2022). Molecular Mechanisms in the Genesis of Seizures and Epilepsy Associated with Viral Infection. Front. Mol. Neurosci. 15, 870868. 10.3389/fnmol.2022.870868 35615063 PMC9125338

[B32] Mcnamara J. O. Scharfman H. E. (2012). “Temporal Lobe Epilepsy and the BDNF Receptor, TrkB,” in Jasper's Basic Mechanisms of the Epilepsies. Editors Noebels Th, J. L. Avoli M. Rogawski M. A. Olsen R. W. Delgado-Escueta A. V. (Bethesda (MD): National Center for Biotechnology Information). 10.1093/med/9780199746545.003.0039 22787630

[B33] Misra U. K. Tan C. T. Kalita J. (2008). Viral Encephalitis and Epilepsy. Epilepsia 49 (Suppl. 6), 13–18. 10.1111/j.1528-1167.2008.01751.x 18754956

[B34] Moore Y. E. Kelley M. R. Brandon N. J. Deeb T. Z. Moss S. J. (2017). Seizing Control of KCC2: A New Therapeutic Target for Epilepsy. Trends Neurosci. 40, 555–571. 10.1016/j.tins.2017.06.008 28803659

[B35] Park H. Poo M.-m. (2013). Neurotrophin Regulation of Neural Circuit Development and Function. Nat. Rev. Neurosci. 14, 7–23. 10.1038/nrn3379 23254191

[B36] Patel D. C. Wallis G. Dahle E. J. Mcelroy P. B. Thomson K. E. Tesi R. J. (2017). Hippocampal TNFα Signaling Contributes to Seizure Generation in an Infection-Induced Mouse Model of Limbic Epilepsy. eNeuro 4, ENEURO.0105-0117.2017. 10.1523/ENEURO.0105-17.2017 PMC5422919 28497109

[B37] Patel D. C. Tewari B. P. Chaunsali L. Sontheimer H. (2019a). Neuron-glia Interactions in the Pathophysiology of Epilepsy. Nat. Rev. Neurosci. 20, 282–297. 10.1038/s41583-019-0126-4 30792501 PMC8558781

[B38] Patel D. C. Wallis G. Fujinami R. S. Wilcox K. S. Smith M. D. (2019b). Cannabidiol Reduces Seizures Following CNS Infection with Theiler's Murine Encephalomyelitis Virus. Epilepsia Open 4, 431–442. 10.1002/epi4.12351 31440724 PMC6698680

[B39] Patel D. C. Wilcox K. S. (2017). “Postinfectious Epilepsy,” in Models of Seizures and Epilepsy. Editors Pitkänen A. Buckmaster P. S. Galanopoulou A. S. Moshé S. L.. Second Edition (Massachusetts: Academic Press), 683–696. 10.1016/b978-0-12-804066-9.00047-x

[B40] Porcher C. Medina I. Gaiarsa J.-L. (2018). Mechanism of BDNF Modulation in GABAergic Synaptic Transmission in Healthy and Disease Brains. Front. Cell. Neurosci. 12, 273. 10.3389/fncel.2018.00273 30210299 PMC6121065

[B41] Rivera C. Li H. Thomas-Crusells J. Lahtinen H. Viitanen T. Nanobashvili A. (2002). BDNF-Induced TrkB Activation Down-Regulates the K+-Cl− Cotransporter KCC2 and Impairs Neuronal Cl− Extrusion. J. Cell. Biol. 159, 747–752. 10.1083/jcb.200209011 12473684 PMC2173387

[B42] Rivera C. Voipio J. Thomas-Crusells J. Li H. Emri Z. Sipila S. (2004). Mechanism of Activity-dependent Downregulation of the Neuron-specific K-Cl Cotransporter KCC2. J. Neurosci. 24, 4683–4691. 10.1523/jneurosci.5265-03.2004 15140939 PMC6729393

[B43] Sasi M. Vignoli B. Canossa M. Blum R. (2017). Neurobiology of Local and Intercellular BDNF Signaling. Pflugers Arch. - Eur. J. Physiol. 469, 593–610. 10.1007/s00424-017-1964-4 28280960 PMC5438432

[B44] Scharfman H. E. Goodman J. H. Sollas A. L. Croll S. D. (2002). Spontaneous Limbic Seizures after Intrahippocampal Infusion of Brain-Derived Neurotrophic Factor. Exp. Neurol. 174, 201–214. 10.1006/exnr.2002.7869 11922662

[B45] Scheirer C. J. Ray W. S. Hare N. (1976). The Analysis of Ranked Data Derived from Completely Randomized Factorial Designs. Biometrics 32, 429–434. 10.2307/2529511 953139

[B46] Shulga A. Thomas-Crusells J. Sigl T. Blaesse A. Mestres P. Meyer M. (2008). Posttraumatic GABAA-Mediated [Ca2+]i Increase Is Essential for the Induction of Brain-Derived Neurotrophic Factor-dependent Survival of Mature Central Neurons. J. Neurosci. 28, 6996–7005. 10.1523/jneurosci.5268-07.2008 18596173 PMC6670975

[B47] Smeal R. M. Fujinami R. White H. S. Wilcox K. S. (2015). Decrease in CA3 Inhibitory Network Activity during Theiler's Virus Encephalitis. Neurosci. Lett. 609, 210–215. 10.1016/j.neulet.2015.10.032 26477780 PMC4867493

[B48] Smeal R. M. Stewart K.-A. Iacob E. Fujinami R. S. White H. S. Wilcox K. S. (2012). The Activity within the CA3 Excitatory Network during Theiler's Virus Encephalitis Is Distinct from that Observed during Chronic Epilepsy. J. Neurovirol. 18, 30–44. 10.1007/s13365-012-0082-5 22328242 PMC4397904

[B49] Steel C. D. Stephens A. L. Hahto S. M. Singletary S. J. Ciavarra R. P. (2008). Comparison of the Lateral Tail Vein and the Retro-Orbital Venous Sinus as Routes of Intravenous Drug Delivery in a Transgenic Mouse Model. Lab. Anim. 37, 26–32. 10.1038/laban0108-26 18094699

[B50] Stewart K.-A. A. Wilcox K. S. Fujinami R. S. White H. S. (2010a). Development of Postinfection Epilepsy after Theiler's Virus Infection of C57BL/6 Mice. J. Neuropathol. Exp. Neurol. 69, 1210–1219. 10.1097/nen.0b013e3181ffc420 21107134 PMC3077028

[B51] Stewart K. A. Wilcox K. S. Fujinami R. S. White H. S. (2010b). Theiler's Virus Infection Chronically Alters Seizure Susceptibility. Epilepsia 51, 1418–1428. 10.1111/j.1528-1167.2009.02405.x 20002148

[B52] Tao W. Chen Q. Zhou W. Wang Y. Wang L. Zhang Z. (2014). Persistent Inflammation-Induced Up-Regulation of Brain-Derived Neurotrophic Factor (BDNF) Promotes Synaptic Delivery of α-Amino-3-hydroxy-5-methyl-4-isoxazolepropionic Acid Receptor GluA1 Subunits in Descending Pain Modulatory Circuits. J. Biol. Chem. 289, 22196–22204. 10.1074/jbc.m114.580381 24966334 PMC4139232

[B53] Umpierre A. D. Remigio G. J. Dahle E. J. Bradford K. Alex A. B. Smith M. D. (2014). Impaired Cognitive Ability and Anxiety-like Behavior Following Acute Seizures in the Theiler's Virus Model of Temporal Lobe Epilepsy. Neurobiol. Dis. 64, 98–106. 10.1016/j.nbd.2013.12.015 24412221 PMC4353639

[B54] Vezzani A. Fujinami R. S. White H. S. Preux P.-M. Blümcke I. Sander J. W. (2016). Infections, Inflammation and Epilepsy. Acta Neuropathol. 131, 211–234. 10.1007/s00401-015-1481-5 26423537 PMC4867498

[B55] Waltl I. Käufer C. Bröer S. Chhatbar C. Ghita L. Gerhauser I. (2018). Macrophage Depletion by Liposome-Encapsulated Clodronate Suppresses Seizures but Not Hippocampal Damage after Acute Viral Encephalitis. Neurobiol. Dis. 110, 192–205. 10.1016/j.nbd.2017.12.001 29208406

[B56] Xu B. Michalski B. Racine R. J. Fahnestock M. (2004). The Effects of Brain-Derived Neurotrophic Factor (BDNF) Administration on Kindling Induction, Trk Expression and Seizure-Related Morphological Changes. Neuroscience 126, 521–531. 10.1016/j.neuroscience.2004.03.044 15183502

[B57] Zhan J. Lin T.-H. Libbey J. E. Sun P. Ye Z. Song C. (2018). Diffusion Basis Spectrum and Diffusion Tensor Imaging Detect Hippocampal Inflammation and Dendritic Injury in a Virus-Induced Mouse Model of Epilepsy. Front. Neurosci. 12, 77. 10.3389/fnins.2018.00077 29497358 PMC5818459

